# Dacron^® ^vs. PTFE as bypass materials in peripheral vascular surgery – systematic review and meta-analysis

**DOI:** 10.1186/1471-2482-8-22

**Published:** 2008-12-19

**Authors:** Stephanie Roll, Jacqueline Müller-Nordhorn, Thomas Keil, Hans Scholz, Daniela Eidt, Wolfgang Greiner, Stefan N Willich

**Affiliations:** 1 Institute for Social Medicine, Epidemiology and Health Economics, Charité University Medical Center, Berlin, Germany; 2 Department of Vascular Surgery, Queen Elisabeth Hospital, Berlin, Germany; 3 Centre for Health Economics and Health System Research, University of Hanover, Germany; 4 Department of Health Economics and Health Management, University of Bielefeld, Germany

## Abstract

**Background:**

In peripheral vascular bypass surgery different synthetic materials are available for bypass grafting. It is unclear which of the two commonly used materials, polytetrafluoroethylene (PTFE) or polyester (Dacron^®^) grafts, is to be preferred. Thus, the aim of this meta-analysis and systematic review was to compare the effectiveness of these two prosthetic bypass materials (Dacron^® ^and PTFE).

**Methods:**

We performed a systematic literature search in MEDLINE, Cochrane-Library – CENTRAL, EMBASE and other databases for relevant publications in English and German published between 1999 and 2008. Only randomized controlled trials were considered for inclusion. We assessed the methodological quality by means of standardized checklists. Primary patency was used as the main endpoint. Random-effect meta-analysis as well as pooling data in life table format was performed to combine study results.

**Results:**

Nine randomized controlled trials (RCT) were included. Two trials showed statistically significant differences in primary patency, one favouring Dacron^® ^and one favouring PTFE grafts, while 7 trials did not show statistically significant differences between the two materials. Meta-analysis on the comparison of PTFE vs. Dacron^® ^grafts yielded no differences with regard to primary patency rates (hazard ratio 1.04 (95% confidence interval [0.85;1.28]), no significant heterogeneity (p = 0.32, I^2 ^= 14%)). Similarly, there were no significant differences with regard to secondary patency rates.

**Conclusion:**

Systematic evaluation and meta-analysis of randomized controlled trials comparing Dacron^® ^and PTFE as bypass materials for peripheral vascular surgery showed no evidence of an advantage of one synthetic material over the other.

## Background

The prevalence of symptomatic peripheral arterial disease in the adult population ranges between 0.6% and 9.2% and increases with age [1,2]. Patients with peripheral arterial disease have an increased risk of cardiovascular morbidity and mortality, showing a similar risk factor profile to patients with other atherosclerotic diseases. In the non-pharmacological treatment of symptomatic peripheral arterial disease, peripheral vascular surgical interventions such as bypass grafting and endarterectomy play an important role [3]. The long-term aim of surgical interventions is to prevent amputation of the limb and to reduce its resulting disability. According to current guidelines, surgical interventions are indicated for individuals with symptomatic disease (claudication), significant functional disability, resistance to exercise or pharmacotherapy, and a reasonable likelihood of symptomatic improvement [4]. Whereas endarterectomy is an option in strictly localised disease, bypass grafting is generally used to circumvent severely stenosed sections of the peripheral arteries.

Different materials can be used for bypass grafting including autologous and homologous grafts from the saphenous vein or the human umbilical vein as well as prosthetic graft materials such as polytetrafluoroethylene (PTFE) or polyester (Dacron^®^) grafts. Most studies so far have shown that autologous vein is superior to prosthetic graft materials in bypass surgery [5-7]. A recent review comparing venous and PTFE bypass procedures reported 5-year primary patency rates of 74% and 39%, respectively [8]. However, almost a third of patients eligible for peripheral bypass procedures do not have suitable veins, making the use of prosthetic materials necessary [9]. Also, due to the high prevalence of cardiovascular co-morbidity, it may be required to keep suitable autologous veins for potential future use in coronary artery bypass grafting. The objective of our systematic review was, therefore, to identify available evidence and compare the effectiveness of the prosthetic bypass materials Dacron^® ^and PTFE in peripheral vascular bypass surgery and to perform meta-analyses, if possible.

## Methods

### Literature search

A trained librarian performed a comprehensive systematic literature search for relevant publications using the following databases: AMED, BIOSIS Previews, CAB Abstracts, CATFILEplus (CATLINE), Cochrane Library – CDSR, Cochrane Library – CENTRAL, Elsevier BIOBASE, EMBASE, EMBASE Alert, ETHMED, GeroLit, GLOBAL Health, HECLINET, IPA, MEDLINE Alert, MEDLINE, NHS-CRD-DARE, NHS-CRD-HTA (INAHTA), NHS-EED, SciSearch, and SOMED. The search terms included "bypass", "revascularization", "artery reconstruction", "graft", "prosthesis", and "material". The search was performed in February 2005 with an update search performed in MEDLINE and CENTRAL (Cochrane Central Register of Controlled Trials) for publications until August 2008. The systematic database search was supplemented by manual search of reference lists of included articles. The inclusion criteria of studies were: (i) randomized controlled trial (RCT) as study design; (ii) comparison of polytetrafluoroethylene (PTFE) or polyester (Dacron^®^) grafts for peripheral vascular bypass surgery; (iii) publication in English or German; and (iv) publication from 1999 to date. We focused on publications of English language to cover the most important and qualitatively high trials therewith (we additionally searched for articles in German for a potential adaption to the situation in Germany). We included studies published in and after 1999 as the purpose of this review was to provide an overview focusing on the present evidence from more recent trials.

Excluded were studies due to the following criteria: (i) case series; (ii) retrospective studies; (iii) studies comparing venous vs. prosthetic graft materials.

### Methodological assessment and endpoints

The methodological quality of relevant publications was assessed using standardized checklists developed by the German Scientific Working Group "Technology Assessment for Health Care". [10] evaluating the selection process of patients, randomization procedure, assessment of outcomes, drop-out rates, and adequate statistical methods. The primary outcome for this review was primary patency, as defined by the authors. According to Rutherford et al. primary patency should be assessed by objective methods such as vascular imaging techniques, palpable pulse, biphasic or triphasic Doppler, segmental limb pressure index, etc. [11]. We here present primary patency rates as defined and reported in each trial. Secondary outcomes were secondary patency, graft infection rates, limb salvage or amputation rates, and perioperative (< 30 days) mortality as presented in each study.

### Statistical analysis

To perform a meta-analysis, study data from accumulated life tables was extracted from each study where available. If failure data was given for one or three months intervals it was combined to yield 6-months interval data for all studies. Time of event (graft failure) or censoring (withdrawals) was thus assigned to the end of each 6-months interval. A Cox proportional hazard model was used to calculate hazard ratios with their 95% confidence intervals (CI) and standard errors (SE) for the independent variable "material". This was done for each study separately. In a random-effect meta-analysis these hazard ratios were then combined with weights according to their standard error (inverse variance method), yielding an overall hazard ratio (with 95% CI). Heterogeneity was tested using the chi-squared Q-statistic and inconsistency was quantified by I^2^. Life table data was also pooled to provide Kaplan-Meier graphs on graft failure events. All analyses were performed with SAS V9.1.3 (SAS Institute Inc., Cary, NC, USA) and Review Manager Version 5.0 (Copenhagen: The Nordic Cochrane Centre, The Cochrane Collaboration, 2008).

## Results

**Figure 1:**
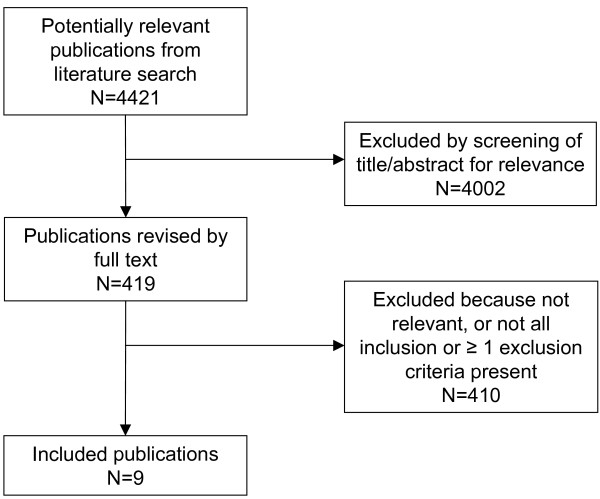
**Literature selection process**.

We identified a total of 4421 publications in the search process. Excluding duplicates and non-relevant publications based on their title and/or abstract resulted in 419 publications for further quality assessment (Fig. 1). Of these, 9 publications were found to be relevant to the research question and fulfilled all inclusion criteria [12-20].

All 9 RCT included in our analysis used primary patency as their main outcome measure. All studies provided a definition for the term patency in their methods section, with primary patency usually meaning unassisted/uninterrupted patency with no follow-up procedures on the bypass assessed by objective methods; one study, however, defined primary patency as assisted primary patency [12]. Seven studies also reported results on secondary patency rates. Five studies present limb salvage or amputation rates, 4 perioperative mortality (<30 days), and 6 graft infection as further outcomes. Most of these endpoints were not defined in detail.

Table 1 gives an overview of study characteristics and main results of all RCT included in our analysis.

**Table 1:** Overview of included studies of Dacron^® ^vs. PTFE as bypass materials in peripheral vascular surgery

**Author**	**Year**	**Indication**	**Site of bypass**	**Intervention**	**Additional intervention**	**Follow-up** **(years)**	N*		**Primary patency** **(N = number of patients or grafts under risk)**	**Superior primary patency**	**Funding**
										
							**Baseline**	**End of study**			
Johnson and Lee [12]	1999	disabling claudication, rest pain or tissue necrosis	femorofemoral, axillofemoral, axillobifemoral	Dacron^® ^vs. PTFE	Aspirin 650 mg/d	5	419	47	At 1, 3, 5 years: - Dacron^®^: 79%, 63%, 50% (N = 125, 73, 32) - PTFE: 77%, 62%, 47% (N = 103, 53, 15)	n.s. (p-value not reported)	n.r.

Robinson et al. [13]	1999	disabling claudication, rest pain or tissue necrosis	femoropopliteal (above-knee and below-knee)	gelatine-sealed Dacron^® ^vs. PTFE	Cephalothin, Heparin, Aspirin	3	108	19	At 1, 2, 3 years: - Dacron^®^: 70%, 56%, 47% (N = 27, 18, 9) - PTFE: 72%, 52%, 52% (N = 33, 16, 10)	n.s. (p = 0.87)	n.r.

Green et al. [14]	2000	superficial femoral artery occlusion	femoropopliteal (above-knee)	collagen-impregnated Dacron^® ^vs. ePTFE	n.r.	5	240	10	At 1, 3, 5 years:: - Dacron^®^: 78%, 65%, 45% (N = 65, 25, 5) - PTFE: 80%, 63%, 43% (N = 66, 21, 5)	n.s. (p-value not reported)	manufacturer of Dacron^®^

Post et al. [15]	2001	indication for artificial graft of at least 20 cm length	femoropopliteal (above-knee and below-knee)	unsealed Dacron^® ^vs. PTFE	Anti-platelet drugs, Heparin or Coumadin	3	194	50	At 3 years (N = grafts under risk): - Dacron^®^: 64% (95%-CI [55%;74%], N = 28) - PTFE: 61% (95%-CI [49%;72%], N = 22)	n.s. (p = 0.89)	manufacturer of Dacron^® ^and PTFE

Prager et al. [16]	2003	aortoiliac occlusive disease	aortoiliac	gelatine-coated Dacron^® ^vs. collagen-coated Dacron^® ^vs. PTFE	Antibiotics, Heparin 70 IU/kg, Fraxiparine 100 mg/kg/d (bid for patients with anastomoses)	8	149	35	At 5, 8 years: - C-Dacron^®^: 89%, 78% (N = 24, 11) - G-Dacron^®^: 92%, 77% (N = 26, 11) - PTFE: 88%, 79% (N = 29, 13)	n.s. (p > 0.8)	n.r.

Robinson and Fletcher [17]	2003	disabling claudication, rest pain or tissue loss	femoropopliteal (above-knee and below-knee)	fluoropolymer-coated Dacron^® ^vs. PTFE	Cephalothin, Heparin, Aspirin	2	129	21	At 6, 12, 24 month: - Dacron^®^: 50%, 36%, 36% (N = 27, 17, 9) - PTFE: 71%, 56%, 47% (N = 43, 28, 12)	PTFE (p = 0.002)	manufacturer of Dacron^® ^and PTFE

Devine and McCollum [18]	2004	occlusive arterial disease (superficial femoral or popliteal artery)	femoropopliteal (above-knee and below-knee)	collagen-coated, heparin-bonded Dacron^® ^vs. PTFE	Aspirin 300 mg/d	5	209	45	At 1, 3, 5 years: - Dacron^®^: 71%, 54%, 46% (N = 70, 45, 20) - PTFE: 62%, 44%, 35% (N = 62, 42, 25)	n.s. (p = 0.05)	n.r.

Eiberg et al. [19]	2006	uni-ilia occlusive disease	femorofemoral	fluoropassivated, gelatine-sealed Dacron^®^ vs. ePTFE	n.r.	2	198	136	At 1, 2 years: - PTFE: 94%, 93% (N = 74, 63) - Dacron^®^: 92%, 87% (N = 87, 73)	n.s. (p = 0.350)	manufacturer of Dacron^® ^and PTFE

Jensen et al. [20]	2007	chronic lower limb ischaemia	femoropopliteal (above-knee)	gelatine-coated Dacron^®^ vs. PTFE	n.r.	2	413	150	At 2 years: - Dacron^®^: 70% (N = 78) - PTFE: 57% (N = 72)	Dacron^®^ (p = 0.002)	manufacturer of Dacron^®^

PTFE: Polytetrafluoroethylene. ePTFE: expanded Polytetrafluoroethylene. IU: international units. d: day. bid: bis in die. n.s.: not significant. n.r.: not reported. CI: confidence interval.

* Number of patients or grafts under risk

Of the 9 included studies, 7 studies showed no significant differences between Dacron^® ^and PTFE regarding primary patency, 1 study showed significantly higher patency rates for Dacron^® ^after 2 years [20], and 1 study showed significantly higher patency rates for PTFE after 2 years [17].

The 7 RCT reporting results on secondary patency yielded results similar to primary patency rates, e.g. no significant differences between the prosthetic materials were found in 6 studies, and 1 study showed significantly higher secondary patency after 2 years with Dacron^® ^grafts. Of the studies reporting results regarding limb salvage or amputation rates, 4 showed no significant difference, whereas 1 showed a significantly better rate for Dacron^® ^grafts. None of the studies found significant differences in terms of graft infection or perioperative mortality (<30 days).

### Meta-analysis

**Figure 2:**
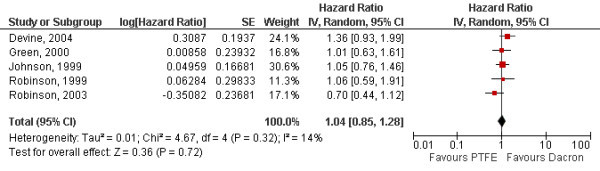
**Forrest plot of estimated hazard ratios on primary patency comparing Dacron^® ^and PTFE for each study and by random-effect meta-analysis (PTFE: polytetrafluoroethylene, SE: standard error, CI: confidence interval)**.

**Figure 3:**
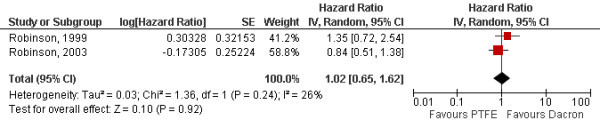
**Forrest plot of estimated hazard ratios on secondary patency comparing Dacron^® ^and PTFE for each study and by random-effect meta-analysis (PTFE: polytetrafluoroethylene, SE: standard error, CI: confidence interval)**.

**Figure 4:**
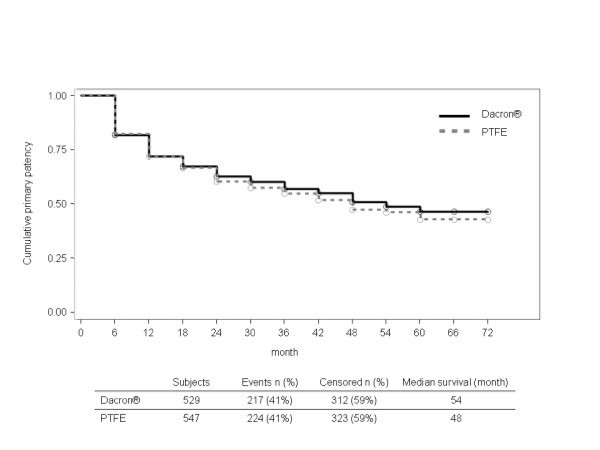
**Survival curves for Dacron^® ^and PTFE on primary patency of pooled data **[12-14,17,18]**(PTFE: polytetrafluoroethylene)**.

**Figure 5:**
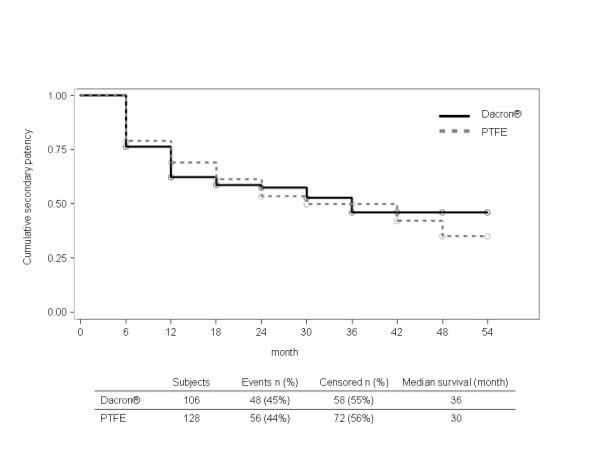
**Survival curves for Dacron^® ^and PTFE on secondary patency of pooled data **[13,17]**(PTFE: polytetrafluoroethylene)**.

Meta-analysis was performed for the comparison of Dacron^® ^vs. PTFE on primary patency for the 5 studies where adequate data was available [12-14,17,18]. Two studies provided also data on secondary patency [13,17]. The combined hazard ratios for PTFE vs. Dacron^® ^were 1.04 (95% CI [0.85;1.28]) for primary patency (Fig. 2) and 1.02 (95% CI [0.65;1.62]) for secondary patency (Fig. 3). There was no significant heterogeneity between the studies (p = 0.32 and p = 0.24). The Kaplan-Meier curves of the pooled data reflect the similar efficacy of the materials regarding both primary and secondary patency (Fig. 4 and Fig. 5).

## Discussion

The present meta-analysis indicated that there are no major differences in primary and secondary patency rates between the two prosthetic graft materials Dacron^® ^and PTFE. Of the 9 included studies, one study showed statistically significant higher patency rates for Dacron^® ^after 2 years [20], 1 study showed statistically significant higher patency rates for PTFE after 2 years [17], while 7 studies showed no statistically significant differences between the two materials regarding primary patency [12-16,18,19].

Our study complements a systematic Cochrane review on femoro-popliteal bypass surgery by Mamode and Scott comparing saphenous vein graft with PTFE or Dacron^®^, human umbilical vein with PTFE, and PTFE vs. Dacron^® ^[21]. However, only one study comparing PTFE with Dacron^® ^in above-knee popliteal grafting was identified [22]. This study by Abbott et al. was published before the search period of our review. It did not show a significant difference regarding primary or secondary patency rates at the 3-year follow-up.

One of the studies in the present review compared gelatine-coated Dacron^® ^vs. collagen-coated Dacron^® ^showing no differences between the study groups [16]. In another study heparin-bonded Dacron^® ^instead of bare Dacron^® ^graft material was used. The issue of different coatings might in itself affect patency outcomes of Dacron^® ^graft. However this question cannot adequately be assessed at the moment due to the lack of data.

Only 5 studies presented data in adequate life table format for performing a meta-analysis. Results of the meta-analysis might change, if more study data could be included, especially data of the trial by Jensen et al. [20] as this was the largest trial to date comparing PTFE vs. Dacron^®^. However, it should be noted that patient recruitment and surgeries of that trial were performed up to 14 years before publication of the results (2 year follow up). For the meta-analysis no raw data with actual times of event or censoring could be used, as only aggregated life table data was available. This will overestimate event and censoring times in both groups, but should not have influenced the comparison of the two graft materials.

In the context of clinical routine care, physicians regularly face decisions as to which alternative treatment strategies to recommend and use. Their decisions should be guided by best possible evidence of previous studies. Hence, systematic reviews of good quality RCT have evolved as important tool of decision support. It is important to note, however, that the RCT included in our systematic review were limited by a number of methodological limitations, such as rather small sample sizes, different methods for determining patency rates, a lack of consideration paid to additional factors that might affect outcomes such as baseline differences between groups, and inadequate interpretations of non-significant results. Reporting of existing baseline differences between the groups in each trial was heterogeneous. Only 4 studies provided information about adjustment for baseline differences as potentially confounding factors [13,14,18,19]. Group differences at baseline may thus have biased the results. In 4 trials adequate sample size calculations were reported [15,18-20]. The other trials may have been too small to detect any differences in the graft materials. In addition, no trial used equivalence testing to show that both graft types were similarly effective. All studies described how primary patency was assessed and defined, and most studies used recommended objective standard methods [11]. However, differences in patency rates between studies might have been affected by unequal assessments, while differences in patency between PTFE or Dacron^® ^grafts within studies should not be affected (assuming same assessment standards for patients receiving PTFE or Dacron^® ^grafts within the trial).

Sources of funding were not specified in 4 trials, with the remaining trials being funded by or having received grants from bypass graft manufacturers, which might introduce bias to the results [23,24]. No explicitly independent/non-manufacturer sponsored trials were found.

In this review only trials published in English or German were considered for inclusion. Even though we feel to cover the most important and qualitatively higher trials with this strategy, this might result in relevant articles and evidence published in other languages being ignored. Results might change if a body of evidence from manuscripts in other languages would be available. Since 4 of the 9 includes trials were conducted in non-English speaking countries (Austria, Germany, and Scandinavian countries), but were published in English, we assume this might not be the case.

## Conclusion

Even in the light of methodological limitations of the included trials, the present meta-analysis and systematic review might offer a basis for clinical decision making in individual patients requiring peripheral vascular surgery. Between the two prosthetic materials PTFE and Dacron^® ^no clear advantage of one over the other could be seen. Further independently funded studies should address the issue of heparin-bonded grafts as well as the identification of subgroups of patients, in which there might be a benefit of one material. Studies should be sufficiently powered to be able to detect differences or equivalence of PTFE and Dacron^® ^grafts.

## Competing interests

The authors declare that they have no competing interests.

## Authors' contributions

SR and DE were responsible for the methodological design of the review, the literature selection and the quality assessment. SR carried out the data extraction, the summary of the findings, the meta-analysis and has participated in the writing of the manuscript. JMN has participated in the writing of the manuscript. TK and WG have revised the manuscript critically. HS advised the review as a clinical expert and has revised the manuscript critically. SW had the overall scientific responsibility and has participated in the writing of the manuscript. All authors read and approved the final manuscript.

## Pre-publication history

The pre-publication history for this paper can be accessed here:


